# A Different Take on Precision Medicine in Nephrology

**DOI:** 10.34067/KID.0000000000000552

**Published:** 2024-08-15

**Authors:** Tessa K. Novick, Deidra C. Crews

**Affiliations:** 1 Division of Nephrology, Department of Internal Medicine, University of Texas at Austin Dell Medical School, Austin, Texas; 2 Division of Nephrology, Department of Medicine, Johns Hopkins University School of Medicine, Baltimore, Maryland; 3 Welch Center for Prevention, Epidemiology and Clinical Research, Johns Hopkins University, Baltimore, Maryland; 4 Johns Hopkins Center for Health Equity, Johns Hopkins University, Baltimore, Maryland; 5 Johns Hopkins O'Brien Center to Advance Kidney Health Equity, Johns Hopkins University, Baltimore, Maryland

**Keywords:** minority health and disparities, nephrology, social health justice

Precision medicine, the concept of providing the right medical intervention for the right person at the right time, has received substantial momentum in recent years.^1^ The *Kidney Precision Medicine Project* and other efforts are using various technologies on kidney biopsy specimens to create a kidney atlas, define disease subtypes, identify and validate biomarkers, and identify targetable disease pathways.^1^ The ultimate goal is to resolve the complexity of the kidney at cellular and molecular levels and improve treatments of kidney diseases.^1^ We are seeing concurrent advances in artificial intelligence; genetic testing; methods to identify novel antigenic targets; xenotransplantation; and medications to treat hypertension, diabetes, and obesity, making it an exciting time in nephrology.

However, we remain saddled with pervasive disparities affecting socially marginalized groups who may be less likely to benefit from recent advances. There is mounting evidence that drivers of disparities include adverse social determinants of health and broader systemic factors.^2^ For example, unstable housing has been associated with increased risk of incident albuminuria, kidney failure, and mortality among patients on dialysis,^3^ and food insecurity has been associated with higher risk of kidney failure and poor adherence to dietary recommendations for people living with kidney disease.^4^ Mental health concerns are common among people with kidney disease and have been associated with poor outcomes, and Black and Hispanic/Latin individuals are more often underdiagnosed and undertreated.^5^ Limited English language proficiency, immigration-related traumatic experiences, low health literacy, and historical redlining promote differential access to medical and mental health care and resources and preparedness to engage in healthy lifestyle behaviors.^2^ To truly individualize care and advance precision medicine in nephrology, it is critical to assess and identify means to intervene on the broader factors that affect kidney health and well-being.

Interventions that address health-related social needs are being tested and implemented across the United States. Hospital-run community garden and teaching kitchens in Portland, Oregon, provide medically tailored produce and gardening classes to people with cardiovascular disease experiencing food insecurity.^6^ In addition to addressing food insecurity by teaching people how to grow their own gardens, these programs promote independence and confidence in the self-management of nuanced nutrition recommendations.

The Seniors Optimizing Community Integration to Advance Better Living with ESRD study provided older adults with kidney failure and low socioeconomic status home visits with an occupational therapist, nurse, and a handy worker to provide ≤$1300 worth of home repairs to support their functioning and was found to be feasible and acceptable and improved activities of daily living disabilities.^7^ Permanent supportive housing is a model that provides low-barrier affordable housing as part of health care and is designed to reduce chronic homelessness and has led to reductions in acute care utilization.^3^

The Accountable Health Communities Model consists of several bridge organizations that provide navigation of community resources for Centers for Medicare & Medicaid beneficiaries with health-related social needs.^8^ Evaluation is ongoing, although findings from 2018 to 2021 demonstrated reductions in emergency department visits.^8^

People with kidney disease experience higher rates of depression than the general population and often face anxiety, post-traumatic stress disorder, and cognitive impairment related to treatments.^5^ Individual mental health is influenced by family mental health, which is affected in the setting of critical illness. A multidisciplinary nephropsychology clinic was recently described, which provided behavioral health interventions to individuals and families at the same time and in the same clinic space as patients' nephrology visits.^5^ Preliminary data demonstrated reduced anxiety and depression symptoms.^5^

Trauma-informed care is a service delivery approach that acknowledges patients' histories of trauma and potential illness-related exacerbations of trauma. Trauma-informed practitioners provide care that recognizes symptoms of trauma and avoids processes and practices that retraumatize patients. In addition to lifetime trauma from adverse childhood experiences, violence, racism, and poverty, starting dialysis serves as a traumatic event for many because many patients develop kidney failure unexpectedly under life-threatening circumstances.^4^ Lifetime trauma has lasting effects on distress tolerance, and people with histories of trauma are more likely to experience debilitating emotional distress when faced with new traumatic situations, such as starting dialysis. People on dialysis have described how dialysis treatments trigger emotional distress and hypervigilance that motivates early termination of sessions.^4^ A trauma-informed approach is a compassionate way to individualize care and mitigate trauma-related emotional distress.

Interventions are needed to increase engagement with populations who have limited health care access. In many US states, undocumented immigrant populations still do not have access to standard care for kidney failure and have inconsistent access to primary and predialysis nephrology care. Identifying and honoring language preferences and offering of complementary and alternative therapies might increase connection with these populations, even in states with policies that limit their health care access. Culturally concordant community health worker and patient navigator interventions show promise in addressing unique immigration-related barriers. A community health worker intervention in Texas provided patient navigation of health care and community resources to recent immigrants from Mexico with proteinuria and kidney disease risk factors.^9^ Most participants lacked health care at enrollment, and everyone in the study made a connection with primary care by the end of the intervention.^9^ Because community health workers usually come from the community they serve, they promote trust and a sense of safety in a health care system that may otherwise be inaccessible.

Rethinking current care delivery models may also be needed. A number of studies have demonstrated the potential benefit of moving interventions from clinics to community locations. For example, conducting home visits, particularly for older adults, might minimize the effect of barriers to attending office visits (*i.e*., transportation or physical limitations). Barbershop-based health outreach has increased in recent years, following the Los Angeles Barbershop Blood Pressure Study, which demonstrated a 21-mm Hg greater reduction in BP among Black hypertensive men who were assigned to a barbershop pharmacist-led intervention when compared with usual care.^10^

At the public and health policy levels, government commitments to address factors that shape social determinants of health, such as affordable housing, fair wages, and access to high-quality education, could have sustained effects on advancing kidney health equity, as would increasing reimbursement for the medical care of individuals with low socioeconomic status.

**Figure 1:**
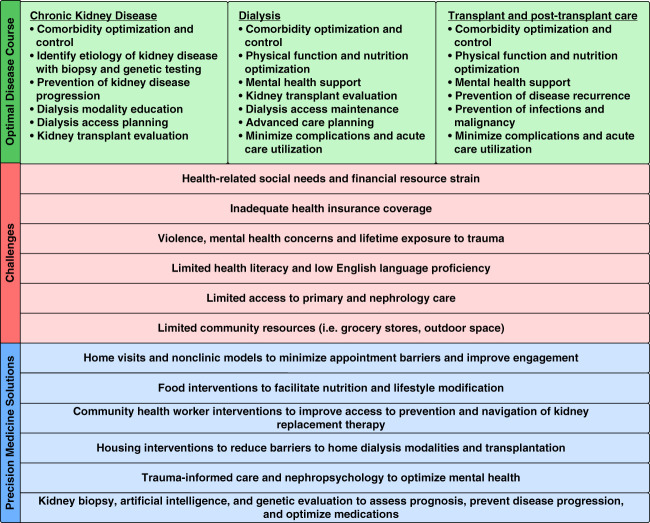
**A broader view of precision medicine in practice.** Numerous challenges prevent socially marginalized groups from benefiting from advances in nephrology. Interventions that address challenges, in conjunction with existing precision medicine practices, are needed.

Patient care decisions surrounding KRT exemplify how this broader view of precision medicine might work in practice (Figure 1). Home visits for older adults with advanced CKD might decrease barriers associated with clinic visits, and thereby increase engagement and education, and highlight elements in the physical environment that need to be addressed. Housing interventions, such as handy-worker repairs, might enable use of home dialysis modalities for people with otherwise unsuitable living conditions. A trauma-informed nephropsychology^5^ component might address the emotional distress associated with the disorienting transition to kidney failure, strengthen family support and understanding, and optimize the person's ability to navigate critical illness. Use of community health workers might facilitate patient navigation of community resources to meet health-related social needs and transplantation evaluation. This would be in conjunction with existing precision medicine practices, such as using genetic testing and histology to understand prognosis, individualize medication regimens, and prevent disease recurrence after transplant.

Special consideration of elements that are unique to each individual and their environment, in conjunction with recent advances in nephrology, has the potential to improve outcomes equitably. Research is needed to understand optimal care practices and inform the policy changes that are necessary to enable creative models of patient-centered care.
